# Recent Advances in the Preparation, Antibacterial Mechanisms, and Applications of Chitosan

**DOI:** 10.3390/jfb15110318

**Published:** 2024-10-27

**Authors:** Kunjian Wu, Ziyuan Yan, Ziyang Wu, Jiaye Li, Wendi Zhong, Linyu Ding, Tian Zhong, Tao Jiang

**Affiliations:** 1 School of Life Science, Zhuhai College of Science and Technology, Zhuhai 519041, China; wkj955@stu.zcst.edu.cn (K.W.); lijiaye@stu.zcst.edu.cn (J.L.); zhongwendi@stu.zcst.edu.cn (W.Z.); dinglinyu359@stu.zcst.edu.cn (L.D.); 2 State Key Laboratory of Marine Environmental Science, College of Ocean & Earth Sciences, Xiamen University, Xiamen 361102, China; yanziyuan@stu.xmu.edu.cn; 3 Faculty of Medicine, Macau University of Science and Technology, Macao 999078, China; tzhong@must.edu.mo; 4 School of Pharmacy, Faculty of Medicine, Macau University of Science and Technology, Macao 999078, China

**Keywords:** chitosan, extraction, antibacterial, mechanism, application

## Abstract

Chitosan, a cationic polysaccharide derived from the deacetylation of chitin, is widely distributed in nature. Its antibacterial activity, biocompatibility, biodegradability, and non-toxicity have given it extensive uses in medicine, food, and cosmetics. However, the significant impact of variations in the physicochemical properties of chitosan extracted from different sources on its application efficacy, as well as the considerable differences in its antimicrobial mechanisms under varying conditions, limit the full realization of its biological functions. Therefore, this paper provides a comprehensive review of the structural characteristics of chitosan, its preparation methods from different sources, its antimicrobial mechanisms, and the factors influencing its antimicrobial efficacy. Furthermore, we highlight the latest applications of chitosan and its derivatives across various fields. We found that the use of microbial extraction shows promise as a new method for producing high-quality chitosan. By analyzing the different physicochemical properties of chitosan from various sources and the application of chitosan-based materials (such as nanoparticles, films, sponges, and hydrogels) prepared using different methods in biomedicine, food, agriculture, and cosmetics, we expect these findings to provide theoretical support for the broader utilization of chitosan.

## 1. Introduction

Chitin, a biopolymer abundantly present on Earth and second only to cellulose in prevalence, is synthesized from N-acetyl-D-glucosamine. This synthesis occurs through β-1,4-glycosidic bonds, which form high molecular polymers that naturally manifest as ordered fibers within the exoskeletons of mollusks, crustaceans, fungi, and insect cuticles [1,2,3]. Chitosan is a derivative of chitin that is produced by partial or complete deacetylation, wherein the acetyl groups in the chitin molecular chain are eliminated, giving rise to amino groups in chitosan. Consequently, chitosan predominantly consists of glycosides linked via β (1→4) bonds of 2-amino-2-deoxy-β-D-glucopyranose (glucosamine) and 2-acetamide-2-deoxy-β-D-glucopyranose units (N-acetylglucosamine). Owing to the protonation of chitosan, it becomes soluble in acidic solutions, such as dilute acetic acid and formic acid, broadening its applicability in various scientific and industrial contexts [4,5,6,7].

**Figure 1:**
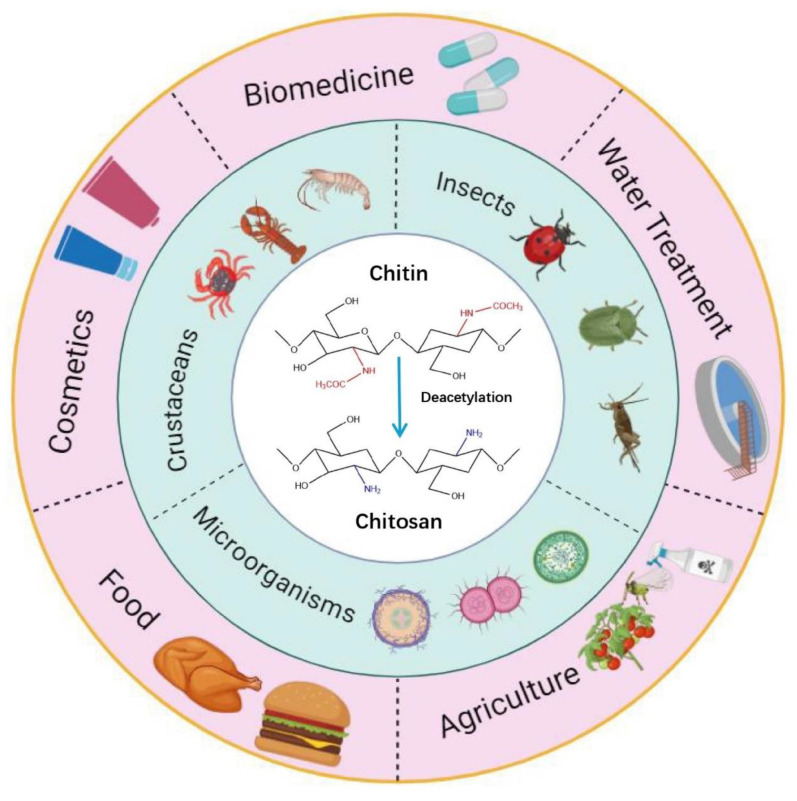
Preparation sources and application fields of chitosan.

Compared with other biopolymers, chitosan exhibits a myriad of advantageous properties, including biocompatibility, biodegradability, and nontoxicity, complemented by its exceptional antibacterial efficacy. These attributes have garnered substantial interest from the scientific and industrial communities since the late 1970s. In the last 5 years, the applications of chitosan and its derivatives have spanned various fields, including the food industry, agriculture, pharmacy, medicine, cosmetics and environmental chemistry [8,9] (Figure 1). Despite making significant progress, current research on chitosan still faces several limitations. For example, chitosan extracted from different sources exhibits variations in molecular weight (Mw), physicochemical properties, and the degree of deacetylation (DD), which can significantly impact its applications [10]. Moreover, the antimicrobial efficacy of chitosan prepared from different sources or methods shows considerable variation under different environmental conditions, potentially related to factors such as pH, Mw, and DD. These complexities prevent chitosan from fully exhibiting its optimal biological functions [11]. Furthermore, there have been substantial recent updates in the manufacturing and applications of chitosan-based biomaterials, including chitosan facial masks. Therefore, this paper reviews the different sources and preparation methods of chitosan, provides an in-depth analysis of the factors influencing its quality and performance, and explores its antimicrobial mechanisms as well as the factors affecting its antimicrobial efficacy. Additionally, this paper summarizes the latest advancements in the applications of chitosan and its derivatives across various fields, noting the new application of chitosan in the form of facial masks in cosmetics. These findings further promote the development and utilization of chitosan.

## 2. Preparation Methods for Chitosan

The solubility, Mw, and DD of chitosan significantly affect its biological properties, and these physicochemical characteristics are closely related to its preparation methods and raw material sources. Specifically, chitosan is generally insoluble in neutral and alkaline environments; however, under acidic conditions, the amino groups in chitosan become protonated, enhancing its solubility in dilute acid solutions. This improved solubility contributes to its widespread use in biomedical applications such as drug delivery systems and wound healing materials, primarily due to its good solubility and biocompatibility. Additionally, the Mw of chitosan typically ranges from 1 × 10^5^ to 1 × 10^7^ Da. A higher Mw leads to increased solution viscosity and mechanical strength, while a lower Mw improves solubility and biodegradability. Lastly, DD refers to the proportion of acetyl groups removed from the chitosan molecule, which is generally greater than 55%. A higher DD indicates a greater density of positive charges on the molecule, thereby enhancing its solubility, antimicrobial activity, and biocompatibility [12,13,14]. Therefore, in the following sections, we will discuss in detail the impact of different raw material sources on the preparation process, as well as the effects on chitosan’s physicochemical properties, Mw, and DD.

### 2.1. Extraction of Chitosan from Marine Crustaceans

The shells of marine crustaceans are rich in chitin. At present, the extraction method of chitin by alkaline heat treatment and the conversion of chitin to chitosan by NaOH are widely used in industry [15,16,17]. Tolesa et al. [17] utilized ammonium-based ionic liquids to extract chitosan from shrimp shells, achieving a yield of 13.4% and a DD of 93%. (Table 1) Moreover, Olafadehan et al. [18] employed an alkaline extraction to obtain chitosan from crab and shrimp shells, resulting in yields of 13.29% and 16.93%, with DD values of 84.2% and 89.73%, respectively. Chitosan extracted from marine crustaceans is characterized by a high molecular weight, high crystallinity, and low solubility. It also possesses a hard texture, making it highly suitable as a thickening agent and material for film formation in various applications within the medical and food industries [19]. However, it is important to note that shells of marine organisms may contain heavy metals that can be harmful to human health, which adds complexity to the extraction process [20,21,22].

### 2.2. Extraction of Chitosan from Insects

Similar to marine crustaceans, chitosan can also be extracted from the exoskeletons and wings of certain insects through alkaline heat treatment. Battampara et al. [23] extracted chitosan from discarded silkworm chrysalises, achieving a DD of 67% and a yield of 18%. In addition, Chae et al. [24] obtained chitosan from crickets, with a DD ranging from 66.54% to 84.98% and a yield of 41.75%. Finally, Amor et al. [25] extracted chitosan from *Blaps lethifera*, *Pimelia fernandezlopezi*, and *Musca domestica*, with yields of 50.0%, 41.6%, and 57.9%, respectively; corresponding DD values were found to be 87.1%, 88.2%, and 84.1%. The chitosan extracted from insects exhibits superior antibacterial properties, biocompatibility, and adsorption capacity compared to that extracted from marine crustaceans, due to its higher Mw, viscosity, and DD. As a result, it is more suitable for applications in areas such as antibacterial treatment, food preservation, and wastewater treatment [22,26].

### 2.3. Extraction of Chitosan Using Microorganisms

Chitosan is widely present in the cell walls of microorganisms, so the extraction of chitosan from fungi and yeast using an alkaline solution is also a common method. Muñoz et al. [27] extracted chitosan with a DD of 73.6% from dry *Aspergillus Niger* mycelium. Similarly, Abdel-Gawad et al. [28] extracted chitosan with a higher DD (83.64%) from *A. niger*. Moreover, Sebastian et al. [29] obtained chitosan with a yield of 13.43 ± 0.3% (*w/w*) and a DD of 94.6 ± 0.9% from *Rhizopus oryzae* NRRL 1526 using microwave-assisted extraction. This method not only enhances the chitosan yield but also contributes to better environmental sustainability. Notably, the research on deacetylating chitin into chitosan using more environmentally friendly deacetylases is gaining increasing attention [20,30]. This method of deacetylation demonstrates high selectivity and controllability, enabling precise regulation to obtain chitosan with desired properties [31]. Rakshit et al. [32] discovered that chitin deacetylase, screened from *Alcaligens faecalis* CS4, can precisely produce chitosan with higher solubility, lower crystallinity, higher antioxidant activity and improved thermal stability.

Microbial fermentation has become a popular method for chitosan production in recent years [33]. By utilizing *Lactobacillus plantarum* and *Bacillus subtilis* [34] for microbial fermentation, chitosan with a DD of 72.90% can be extracted from shrimp waste. However, using *Lactobacillus delbrueckii*, *Bifidobacterium lactis*, and *A. niger* [35], chitosan with a DD greater than 78% can be successfully extracted from shrimp shells. Moreover, the chitin-rich *F. velutipes* was inoculated with *A. niger* to produce high-purity chitosan, yielding 56.3% ± 0.47%, and achieving a DD of 99.2% ± 1.07% [36]. Although microbial fermentation achieves resource recycling, the demanding cultivation conditions pose challenges for industrialization. In addition, the chitosan extracted through microbial fermentation has a low Mw, moderate viscosity, and high DD (range 70–95%). These properties significantly enhance the biocompatibility and solubility of chitosan, making it more advantageous for applications in the food and medical industries [21,22,37].

## 3. Antimicrobial Activity

### 3.1. Mechanisms

**Figure 2:**
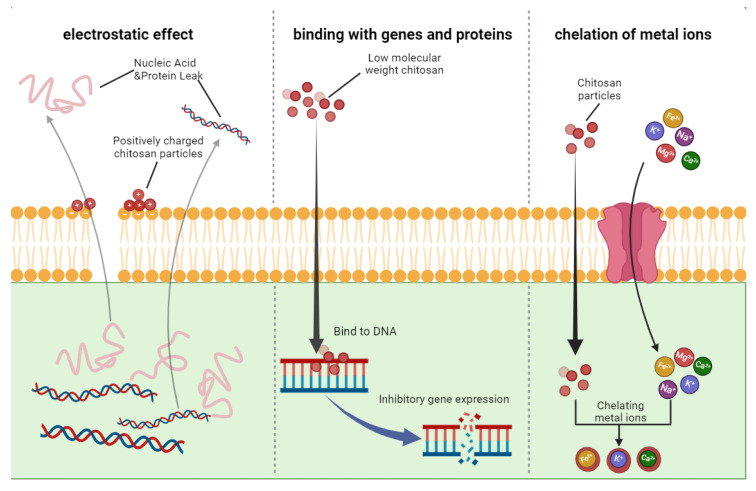
Antibacterial mechanism of chitosan.

The antibacterial mechanism of chitosan against bacteria can be summarized as the disruption of bacterial cell walls, inhibition of bacterial mRNA and protein synthesis, as well as chelation with metal ions [38,39]. Mechanistically, when the pH value is below 6, the amino group at the C-2 position of chitosan becomes positively charged, leading to an interaction between chitosan and bacterial cells. This interaction alters the integrity of cell walls and results in DNA attachment, ultimately inhibiting DNA replication and causing bacterial cell death [40] (Figure 2). The antifungal properties of chitosan are demonstrated by its effective inhibition of spore germination, germ tube elongation, and radial growth in fungi. These effects may be attributed to the regulatory changes induced by chitosan during host–fungi interactions [40].

As the DD in chitosan increases, there is a corresponding increase in the protonation level of its amino groups, which subsequently enhances its antibacterial efficacy [41,42]. For instance, a comparative analysis of chitosan with DD levels of 75%, 85%, and 95% revealed a clear positive correlation between DD levels and antibacterial activity against *Escherichia coli* and *Staphylococcus aureus*; specifically, higher DD levels were associated with increased antibacterial potency [43].

Low Mw chitosan has the ability to effectively penetrate bacterial cell walls, leading to the inhibition of gene expression and protein synthesis in microorganisms [20,44]. Moreover, it can also penetrate the cell wall of *Candida albicans* and hinder the expression of genes associated with cell wall functions (such as ALS2, pga45, and ACE2) as well as transport genes (like MDR1 and CDR1), thereby exhibiting its antifungal properties [45]. The inhibitory impact observed is closely linked to the Mw of chitosan. Conversely, studies have revealed that higher Mw chitosan demonstrates significant inhibitory effects on pathogenic *Fusarium oxysporum* f. sp. and *Alternaria solani* when compared to lower Mw chitosan [46].

When the pH value exceeds the pKa value of chitosan (6.2~6.5), the amino groups on chitosan will tend to chelate with metal ions (Na^+^, Ca^2+^, Mg^2+^, Fe^2+^ and Zn^2+^), thereby inhibiting the uptake of essential elements for cell growth and achieving antibacterial effects [47]. However, during this process, there is a noticeable decrease in the degree of protonation of amino groups within chitosan [7,9,48]. So, chelation of metal ions is not the primary antimicrobial mechanism of unmodified chitosan. Instead, in chitosan modified with vinylsulfonic acid sodium salt or carboxymethyl groups, the vinylsulfonic acid sodium and carboxymethyl groups can chelate with metal ions, thereby enhancing the antimicrobial activity of chitosan [49].

### 3.2. In Vitro Effects

The antimicrobial activity of chitosan varies against different microbes, which can be attributed to the target microbe and its growth state, as well as the concentration, pH, zeta potential, molecular weight, and acetylation degree of chitosan [50]. Aguayo et al. [51] employed chitosan nanoparticles combined with tripolyphosphate to detect the antibacterial effect of *Pseudomonas* sp. isolated from milk samples. The findings revealed that the chitosan nanoparticles with tripolyphosphate exerted a potent antibacterial effect. (Table 2) Nahrawy et al. [52] prepared chitosan/phosphosilicate/Al₂O₃ nanosheets to enhance antibacterial activity. These nanosheets exhibited significantly stronger antibacterial activity than chitosan alone, and their antibacterial efficacy increased with increasing concentrations of Al₂O₃. In addition, to address the issue of bacterial resistance, Facchinatto et al. synthesized a chitosan/cyano-substituted poly (*p*-phenylene vinylene) nanocomposite (chitosan/CNPPV NCPs) with photo antibacterial activity. This nanocomposite exhibits high inhibitory activity against *E. coli* and *S. aureus*, with the strongest inhibitory effect observed under blue light irradiation [53]. However, the solubility of chitosan is low in neutral and alkaline solutions, which limits its antibacterial effectiveness. The antimicrobial capability of chitosan can be enhanced by preparing its derivatives through methods such as carboxymethylation, alkylation, and quaternization [9,54]. For example, compared with unmodified chitosan, carboxymethyl chitosan and its metal composite materials exhibit superior antibacterial effects against *E. coli*, *Pseudomonas aeruginosa A*, *Pseudomonas aeruginosa B* and *Klebsiella* sp. [55]. Furthermore, N-alkylated chitosan obtained through alkylation treatment exhibits significantly higher antibacterial activity against *E. coli*, *P. aeruginosa*, *S. aureus*, and *Bacillus cereus* than unmodified chitosan [56]. Moreover, quaternized chitosan nanofibers containing vanillin effectively inhibit the growth of *E. coli*, *S. aureus*, and *C. albicans* strains [57]. The quaternized chitosan-dialdehyde cellulose composite sponges exhibit superior performance in suppressing both *S. aureus* and *E. coli*, making them suitable for wound care and emergency hemostasis [58]. Fully deacetylated quaternary chitosan demonstrates significant inhibitory effects against bacteria (*S. aureus* and *E. coli* O157: H7) and fungi (*C. albicans* and *Aspergillus flavus*), particularly showing remarkable efficacy against *C. albicans* [59].

## 4. Applications

### 4.1. Biomedicine and Pharmacotherapy

The preparation of chitosan-based biodegradable dressings or fillers can be utilized for wound healing and repair [60,61]. The role of chitosan in accelerating wound healing is attributed not only to its ability to stimulate cell proliferation and enhance the production of hyaluronic acid and collagen but also to its structural similarity to components of the extracellular matrix, particularly glycosaminoglycans. This similarity enhances the interaction between chitosan and cells, promoting cell colonization and stimulating cell differentiation [62,63]. Du et al. [64] designed a hemostatic chitosan sponge by combining 3D-printed microfiber extraction, freeze-drying, and surface-active modification, characterized by extensively interconnected microchannels. This microchannel chitosan sponge demonstrated superior procoagulant and hemostatic capabilities in both standard and heparinized rat and pig liver perforation wound models compared to conventional gauze, gelatin sponges, and Celox™ (Table 3). The introduction of alkylated chitosan enhances its hydrophobicity, which can limit fluid exchange between the external environment and the wound tissue. At the same time, it forms a sealing barrier on the wound surface to prevent the penetration of airborne bacteria and maintain gas exchange, thus significantly promoting the wound healing [65,66]. Recently, Sun et al. [67] developed an alkylated chitosan and diatom biosilica hemostatic composite sponge (AC-DB) using a freeze-drying method. The in vivo evaluation showed that the clotting time of the AC-DB sponge was 106.2 s, and the blood loss was 328.5 mg, demonstrating excellent coagulability. Sponges prepared using the freeze-drying method exhibit excellent water absorption and breathability, with a relatively fast degradation rate, showing outstanding performance in the field of wound healing [10]. Moreover, Chi et al. [68] innovated a biomass chitosan microneedle array patch using a blending method, designed for enhancing wound healing and facilitating intelligent responsive drug delivery. The results indicated that the chitosan microneedle array patch not only accelerated wound healing in severe infection wound models but also promoted anti-inflammatory effects, collagen deposition, angiogenesis, and tissue regeneration during the wound closure process. The blending method, which combines chitosan with other materials, can significantly enhance the mechanical properties of the resulting material [14]. Kumar et al. [69] were the first to synthesize agarose composites embedded with chitosan–silver nanoparticles using chemical cross-linking, with glutaraldehyde as the cross-linking agent. This material possesses an impressive swelling ratio, exemplary hemocompatibility, and favorable biocompatibility with HeLa, MiaPaCa-2, and HEK cells. Chemical cross-linking forms a network structure using cross-linking agents, which greatly enhances the mechanical strength and stability of the materials, making them suitable for long-term applications [70]. To enhance the antibacterial and antioxidant capabilities at wound sites, a complex of 2-hydroxypropyltrimethyl ammonium chloride (HTCC) and *p*-coumaric acid (*p*-CA) was synthesized. This complex was then used to prepare quaternized chitosan nanoparticles with p-coumaric acid (HTCC-CA NPs) through ionic gelation with sodium tripolyphosphate. The in vitro test demonstrated that HTCC-CA NPs exhibited high antibacterial activity (with consistent MIC and MBC values), excellent antioxidant capacity (radical scavenging rate >65%), and low cytotoxicity, making them a promising biomedical material for promoting wound healing [71]. Biomaterials prepared by the ionotropic gelation method form a three-dimensional gel network through ionic cross-linking, exhibiting good flexibility and biocompatibility, with a moderate degradation rate [72]. In addition, researchers have developed a chitosan vitamin C–lactic acid membrane using the freeze–gelation method. This membrane exhibited superior biocompatibility with NIH3T3 cells, thereby highlighting its potential utility in future skin tissue engineering endeavors [73]. Furthermore, Saravanan et al. [74] developed a chitosan/glycerophosphate/graphene oxide hydrogel specifically for bone tissue repair. Under osteogenic conditions, this hydrogel significantly promoted the osteogenic differentiation of mouse mesenchymal stem cells, demonstrating its potential application in bone tissue engineering.

Chitosan exhibits significant promise in the field of drug delivery due to its biocompatibility, biodegradability, low toxicity, non-adhesion, and non-irritability. Furthermore, chitosan and its derivatives are pH sensitive and can achieve rapid drug release under acidic conditions and slow drug release under alkaline conditions. This property makes chitosan an excellent material for controlled drug release [75,76,77]. Yu et al. [78] designed spherical nanoparticles that encapsulated curcumin and quercetin using pH-sensitive octenyl succinyl chitosan. These nanoparticles are highly sensitive to weakly acidic conditions (pH 6.0) and exhibit a faster release rate, while they have significant solubility and biocompatibility under physiological conditions (pH 7.4), thereby improving their therapeutic effect and cellular uptake. Taherian et al. [79] formulated magnetic chitosan nanoparticles loaded with black pomegranate peel extract using the freeze-drying method, and the results showed that these nanoparticles exhibited significant drug-loading efficiency and release rate in a simulated tumor environment and had no toxic effect on normal cells. In an acidic environment, chitosan is positively charged, which has a guiding impact on negatively charged drug molecules or the cell surface, playing a protective and controlled release role in drug delivery [80]. Lu et al. [81] prepared chitosan/poly (lactic-co-glycolic acid) nanoparticles loaded with paclitaxel through electrostatic adherence. The release rate of nanoparticles varied with pH and showed accelerated release at pH 5.5, which was conducive to rapid drug delivery in tumor tissues. The electrostatic adherence method typically involves attaching nanoparticles to surfaces with opposite charges, offering advantages such as multifunctionality, good biocompatibility, favorable mechanical properties, and controlled degradation characteristics. This makes it widely applicable in the preparation of functional coatings and drug carriers [82]. Niu et al. [83] used the freeze-drying method to copolymerize chitosan with N-vinylcaprolactam to encapsulate doxorubicin and added cell-penetrating peptides to enhance drug penetration into cells. This new drug delivery system significantly inhibited the proliferation of cancer cells and had lower off-target effects than did doxorubicin alone. In addition, some researchers have also prepared chitosan/polylactide glycolic acid/docetaxel loaded nanoparticles [84], galactosylated chitosan/graphene oxide/doxorubicin loaded nanoparticles [85] and chitosan/paclitaxel/dextran sulfate/chitosan-5-fluorouracil double-loaded nanoparticles [86]. All these drug-loaded nanoparticles can control drug release at the tumor site and have obvious inhibitory effects on tumor cells. The cationic properties of chitosan also make it an effective carrier of nucleic acids [87]. Furthermore, Hajam et al. [88] used an anionic gelation method to encapsulate the mRNA molecules of H9N2 HA2 and M2e influenza proteins in chitosan nanoparticles to effectively penetrate the mucosal barrier and deliver them to antigen-presenting cells. In addition, chitin/chitosan nanocrystals, which retain their original favorable characteristics while exhibiting enhanced mechanical strength, antimicrobial activity, and functionalization capabilities, are gradually gaining attention [89,90,91]. Dos Santos et al. [92] synthesized a dual-layer composite hydrogel membrane by 3D printing, which incorporated lipid nanoparticles, grape seed extract, simvastatin, and chitin nanocrystals. The results indicate that the addition of chitin nanocrystals significantly improves the mechanical properties of the composite membrane and extends the drug release period to over 24 days, promoting periodontal tissue regeneration and reducing inflammation. Additionally, Jin et al. [93] prepared chitosan nanocrystals through a solid-phase aging method and combined them with alginate to produce a composite hydrogel for drug release. Compared to the common chitosan hydrogel, the chitosan nanocrystal hydrogel exhibited higher crystallinity and mechanical strength, which may enhance drug release efficiency. Finally, Hrapovic et al. [94] utilized persulfate oxidation to prepare a chitin nanocrystal containing antimicrobial peptide components, which can regulate the antimicrobial performance of the material through different peptide chains and thus achieve the targeted cell delivery.

Due to its exceptional biocompatibility, antimicrobial characteristics, and capacity to enhance osteoblast differentiation, chitosan finds extensive application in the fabrication of diverse scaffolds [95,96]. Kandil et al. [97] developed a chitosan–hydroxyapatite–lignin hybrid composite scaffold using lyophilization technology for the postoperative management of osteosarcoma. This scaffold exhibits an enhanced water absorption capacity and promotes cell adhesion and proliferation, thereby facilitating the repair and reconstruction of bone tissue. In addition, Zhang et al. [98] also fabricated a chitosan–sodium alginate/bioactive glass composite cartilage scaffold using the freeze-drying method. This scaffold demonstrated excellent mineralization properties and cytocompatibility in vitro, making it suitable for the treatment of cartilage injuries. However, compared to the aforementioned scaffolds, chitosan hydrogel scaffolds offer superior advantages for nutrient transport due to their porous architecture [99]. Lin et al. [100] prepared a composite hydrogel scaffold based on carboxymethyl chitosan using a chemical crosslinking method, which exhibited excellent cell adhesion and biocompatibility. This scaffold can provide outstanding mechanical support for wounds and promote the regeneration and repair of cartilage tissue. Furthermore, Phatchayawat et al. [101] successfully prepared a 3D hydrogel scaffold that could support the proliferation of human mesenchymal stem cells by chemically cross-linking bacterial cellulose, chitosan, alginate, and gelatin. After culturing, the scaffold contained 6.26% glycosaminoglycans and 6.71% collagen. The 3D-printing method creates three-dimensional objects by depositing materials layer by layer, allowing precise control over the shape, structure, and internal porosity of the materials. This makes it particularly suitable for applications in tissue-engineering scaffolds [102]. In addition, the composite scaffold prepared by freeze-drying a mixture of chitin nanocrystals and chitosan exhibited excellent biocompatibility, water absorption, mechanical properties, and a porous structure (porosity >80%). This scaffold significantly promoted the adhesion and proliferation of osteoblast cells, demonstrating great potential for applications in bone-tissue engineering [103].

**Table 3:** Application of chitosan in biomedicine.

Applications	Forms	Drugs/Other Ingredients	Symptoms/ Diseases	Beneficial Effects	Ref.
Wound healing	Sponge	-	Wound infection	Showed superior coagulant and hemostatic ability	[64]
		Diatom biosilica			[67]
	Hydrogel	Vascular endothelial growth factor		Promoted wound healing and intelligent reactive drug delivery	[68]
	Nanoparticles	Nano silver		Showed high swelling rate, blood compatibility and good biocompatibility	[69]
		*p*-coumaric acid		Exhibited high antibacterial activity, antioxidant activity, and low cytotoxicity	[71]
	Membrane	Vitamin C and lactic acid		Improved biocompatibility with cells	[73]
	Hydrogel	Graphene oxide		Repaired bone tissue	[74]
Drug delivery and controlled release	Nanoparticles	Curcumin and quercetin	Inflammation	Improved therapeutic outcomes and cellular uptake	[78]
		Black pomegranate peel extract	Breast cancer	Possessed remarkable drug loading efficiency and drug release rate	[79]
		Paclitaxel		Promoted rapid drug delivery in tumor tissue	[81]
		Doxorubicin	Triple-negative breast cancer	Inhibited the proliferation of cancer cells	[83]
		Docetaxel	Colon cancer	Controlled the release of drugs at the tumor site	[84,85,86]
		Doxorubicin	Liver cancer		
		Paclitaxel and 5-fluorouracil			
		Influenza protein	Avian influenza	Improved drug utilization	[88]
	Hydrogel	Lipid nanoparticles, grape seed extract and simvastatin	Periodontitis	Promoted periodontal tissue regeneration and reduced inflammation	[92]
		Bovine serum albumin	-	Enhanced mechanical performance	[93]
	Nanocrystals	Antimicrobial peptide		Controlled the release of drugs at the targeted site	[94]
Bone-tissue scaffold	Scaffold	Hydroxyapatite and lignin	Osteosarcoma	Promoted the repair and reconstruction of bone tissue	[97]
		Bioactive glass	Cartilage injury	Exhibited excellent mineralization performance and cell compatibility	[98]
	Hydrogel scaffold	-	Cartilage injury	Provided excellent mechanical support for wounds	[100]
		Bacterial cellulose, alginate and gelatin	Bone-tissue injury	Provided nutrients to the injured tissue	[101]
	Scaffold	Chitin nanocrystals		Promoted the proliferation of osteoblasts	[103]

### 4.2. Water Treatment

Chitosan, which is enriched with abundant amino and hydroxyl functional groups, can form hydrogen bonds, electrostatic interactions, and other adsorption mechanisms with heavy metal ions and organic pollutants in water, thus efficiently purifying contaminated water [104,105,106]. Zhang et al. [107] synthesized a novel polyacrylic acid-grafted chitosan/biochar composite that had a high adsorption capacity for ions such as Cu^2+^, Zn^2+^, Ni^2+^, Pb^2+^, Cd^2+^, Mn^2+^, Co^2+^, and Cr^3+^ (Table 4). To enhance the selectivity and adsorption capacity for metal ions, a bifunctional chitosan derivative called EDTA-carboxymethyl chitosan (EDTA-CMC) was synthesized by linking EDTA with carboxymethyl groups onto chitosan. In adsorption studies of Cu²⁺, EDTA-CMC exhibited a maximum adsorption capacity of 112.44 mg/g [108]. Moreover, Sessarego et al. [109] developed phosphonium-crosslinked chitosan using phosphonium salt (tetrahydroxymethyl) phosphonium sulfate and functionalizing chitosan for the adsorption of Cr (VI) in wastewater. This phosphonium-crosslinked chitosan possesses a superior adsorption capacity and is more versatile for wastewater treatment across a broader pH spectrum than unmodified chitosan. In addition, chitosan can also serve as a flocculant to remove microorganisms from water, thereby improving water quality. Li et al. [110] reported that 3-chloro-2-hydroxypropyl trimethyl ammonium chloride chitosan-modified clay (CTCMC) can effectively inhibit algal blooms in seawater. The removal efficiency was approximately 80% when the concentration of CTCMC reached 20 mg/L. Furthermore, chitosan can also be used as a raw material to react with lactic acid to prepare chitosan lactate (CSS), which is used to treat microalgae in the ocean. The CSS solution has great ability in recovering microalgae, and the recovery rate of microalgae can reach 90% after 120 min under the optimal conditions of pH = 10 [111]. Moreover, chitosan and inorganic salts such as ferric chloride or aluminum sulfate can produce a synergistic effect to flocculate harmful algal blooms in water, resulting in a flocculation efficiency of over 80%, which is significantly greater than that of chitosan used alone [112].

### 4.3. Agricultural Applications

Chitosan possesses non-toxic, biodegradable, and antibacterial properties. Consequently, in crop production, chitosan can serve as a drug carrier to enhance the delivery properties of pesticides and synergize with chemical pesticides to improve their efficacy [113,114]. To improve the inhibitory effect on agricultural pathogens, Tomke et al. [115] prepared Fe3O4@chitosan AgNP nanoparticles by adding nanosilver. The results showed that these nanoparticles not only exhibited significant antibacterial activity against various agricultural pathogens (including *Colletotrichum coccodes*, *Aspergillus niger*, and *Pyricularia* sp.) but also catalyzed the reduction in the anthropogenic pollutant 4-nitrophenol (Table 5). Moreover, Sharma et al. [116] encapsulated the pesticides spinosad and permethrin in chitosan nanoparticles. Compared with those of nonencapsulated pesticides, the insecticidal and bactericidal effects of nanopesticides were significantly improved. Therefore, the wide application of chitosan may not only reduce people’s dependence on chemical pesticides but also reduce environmental pollution. In addition, Campos and associates [117] showed that chitosan/β-cyclodextrin was used as the substrate to encapsulate carvacrol and linalool, and the anti-mite effect was bioassayed. The results showed that these nanoparticles could repel, kill mites and inhibit their oviposition. Notably, nanoencapsulated carvacrol and linalool showed significantly greater efficacy in killing mites and inhibiting oviposition, while free carvacrol and linalool had better anthelmintic effects.

Meanwhile, we cannot ignore the other applications of chitosan in agriculture. It is a growth regulator that not only promotes plant growth but also promotes seed germination. Hartoyo et al. [118] sprayed rice seeds with chitosan nanocomposites to observe their effects on growth. The results indicate that spraying an appropriate amount of chitosan nanocomposite material can promote rice growth during both germination and greenhouse testing. Under optimal growth conditions, the birth rate and growth rate reached 16.44% and 65.74%, respectively (Table 6). In addition, copper (II) complex in chitosan/alginate microcapsules can also be used for regulating plant growth and providing trace amounts of calcium and copper sources for plant growth. As the concentration of Cu (II) complex increases, plant development accelerates, but higher concentrations can inhibit plant growth [119]. Furthermore, Du et al. [120] treated ungerminated seeds with modified oxidized chitosan and found that it had a good promoting effect on seed germination, with a germination rate of 74.5%. It is also a soil amendment that can not only remove heavy metals to improve soil characteristics and remediate contaminated soil but also increase crop yields. Xu et al. [121] prepared a chitosan-modified scrap iron-filing composite to stabilize soil contaminated by the heavy metal arsenic. The results showed that the stabilization efficiency of the heavy metal arsenic reached 73.98% at pH 5~6. Moreover, chitosan significantly enhanced the electron transfer rate and photosynthesis rate in lettuce leaves, resulting in increased lettuce yield. This suggests its potential application in the sustainable production of lettuce [122].

### 4.4. Food

As a new type of film packaging raw material, chitosan has exceptional biocompatibility, antibacterial activity, and biodegradability, which makes it widely used in the food industry [123,124]. From basic chitosan antibacterial films to those films incorporating additives such as metal nanoparticles, graphene, fullerenes, and plant extracts, each type of film has its own unique synthesis method and biological properties [125]. Soltani et al. [126] prepared two types of nanocomposite films derived from nanocellulose: one based on starch–chitosan and the other based on gelatin–chitosan. The results indicate that increasing the nanocellulose content improves the elongation at break and the transparency of the film. Conversely, increasing the chitosan content enhanced the food preservation capabilities and opacity of the films (Table 7). In addition, gelatin-based films exhibit excellent transparency, tensile strength, and elongation at break. However, starch-based films are superior in terms of food preservation. To improve the antioxidant capacity of chitosan-based films and maintain the freshness of vegetables and fruits, active ingredients from discarded blueberry and blackberry residue extracts can be incorporated into the chitosan matrix to create a composite film. This film, which is rich in anthocyanins, changes color with pH, allowing for the determination of food spoilage. Notably, the antioxidant capacity of the film is directly proportional to the content of fruit pomace extract, with blackberry pomace demonstrating a particularly strong antioxidant capacity [127]. Furthermore, mango leaf extract can also be added to chitosan-based films. The results indicated that increasing the concentration of mango leaf extract can increase the film thickness, reduce the water content, and enhance the antioxidant capacity. Compared with that of commercial polyamide/polyethylene films, the antioxidant capacity of 5% composite films is 56% greater, indicating the enormous potential of this composite film as a food preservation active packaging material [128]. Moreover, chitosan as a food additive also functions as an excipient and preservative [129]. Since 1980, lactobacilli have been allowed by the US Food and Drug Administration to be used as preservatives in the food industry. However, direct use of Lactobacillus peptides in food can lead to uncontrolled interactions, electrostatic repulsion, and degradation of various food ingredients. To address this issue, Khan et al. [130] utilized chitosan monomethyl fumaric acid nanoparticles loaded with Lactobacillus peptides. The results demonstrated that these nanoparticles prominently reduced the bacterial count in orange juice after 48 h. In addition, to further enhance the preservation effect, preservative paper containing 2-hydroxyphenylacetic acid-g-chitosan can be prepared by a coupling reaction, and honey peach is included to observe the preservation effect. The results showed that when honey peach was stored at 20 ± 5 °C and 40%~60% humidity, the preservation effect was the best [131]. Chlorine dioxide has a strong antioxidant capacity and can be used for the preservation of fruits and vegetables, but the instability of chlorine dioxide limits its application. Wu et al. [132] reported that the synergistic combination of carboxymethyl chitosan citrate and chlorine dioxide enhanced the preservative effect on fruits and vegetables and improved the utilization rate of chlorine dioxide. Additionally, the food industry is in urgent need of a new environmentally friendly disinfectant, and quaternized chitosan products may fulfill this requirement. Therefore, Cohen et al. [133] developed quaternary dimethyl-(alkyl)-ammonium chitosan derivatives (QACs) for the preservation of spinach. Antimicrobial activity tests on fresh spinach leaves demonstrated a significant reduction in the number of pathogens present on the spinach.

### 4.5. Cosmetics

Chitosan nanoparticles are significantly applied in the fields of cosmetics and transdermal drug delivery systems. They enhance drug penetration into and through the skin, improving drug efficacy and tolerance in the human body. Furthermore, studies have demonstrated that chitosan nanoparticles are nontoxic to human dermal fibroblasts and exhibit good cellular internalization [134]. Hyaluronic acid is widely used in the cosmetics industry for its physiological functions of maintaining skin moisture and smoothness, anti-wrinkle, aesthetic, health care, and repairing the skin (Table 8). However, its high Mw prevents it from penetrating deep into the skin and thus preventing it from exerting its effects. Therefore, researchers used quaternized cyclodextrin-grafted chitosan nanoparticles loaded with hyaluronic acid to enable them to penetrate deep into the skin and exert their effects [135]. In addition, chitosan also possesses properties such as water resistance and UV absorption, making it suitable for sun protection applications. Ntohogian et al. [136] developed a sunscreen based on chitosan nanoparticles loaded with annatto, ultra-filtrated annatto, saffron, and ultra-filtrated saffron. This sunscreen has good preservation and low toxicity, and the sun protection factor (SPF) ranges from 2.15 to 4.85.

Facial masks are the most common kind of skin care product. It has the functions of skin moistening, anti-aging, and so on. Because it is easy to use, the demand for it is increasing [137]. Chitosan has excellent film-forming ability and antioxidant properties, so some scholars have used 1% (*w/w*) chitosan, 1% (*w/w*) annatto powder, 5% (*w/w*) vitamin C and 1% (*w/w*) glycerol to prepare an active anti-aging film. The results show that this film has excellent anti-aging ability and is not toxic to cells, so it has potential application in the field of anti-aging facial masks [138]. In addition, to enhance their anti-aging ability, lemon grass essential oil chitosan facial masks can be prepared by adding lemon grass essential oil to chitosan films. Lemongrass essential oil has strong antioxidant and antibacterial properties. The chitosan facial mask containing 1.5% lemongrass essential oil has the same ability to inactivate intracellular reactive oxygen species as the antioxidant N-acetyl-L-cysteine and has no toxic risk to cells [139]. Furthermore, inhibiting the formation of melanin can also prevent skin aging. *Phyllanthus emblica* extract can inhibit tyrosinase and prevent melanin production, thus achieving the goal of preventing skin aging. Lin et al. [140] mixed polyvinylpyrrolidone and chitosan with *Phyllanthus emblica* to make a dry facial mask to prevent the formation of melanin. It is worth noting that this dry facial mask of *Phyllanthus emblica* not only has an amazing inhibitory effect on tyrosinase of 99.53 ± 0.45% but can also limit the activity of inflammatory factors.

**Table 8:** Applications of chitosan in cosmetics.

Application	Forms	Beneficial Effects	Ref.
Transdermal absorption	Chitosan nanoparticles	Enhanced the penetration of drugs in the skin, improved the activity and tolerance of drugs in the human body	[134]
	Chitosan nanoparticles loaded with hyaluronic acid	Exerted carrier function to deliver effective ingredients	[135]
Sunscreen	Sunscreen chitosan nanoparticles	Absorbed UV to achieve sun protection effect	[136]
Facial mask	Anti-aging chitosan active film	Showed excellent anti-aging ability and nontoxic to cells	[138]
	Lemongrass essential oil chitosan mask	Enhanced anti-aging ability	[139]
	*Phyllanthus emblica*/chitosan dry mask	Inhibited melanin and prevented skin aging	[140]

## 5. Perspectives and Conclusions

Chitosan, a natural, high molecular material, is characterized by its outstanding biocompatibility and bioactivity, and finds extensive applications in the fields of medicine, food, cosmetics, water treatment, and agriculture [141]. While the preparation technology of chitosan is already quite advanced, addressing the urgent issue of increasing its yield and enhancing its performance remains a priority. Compared to traditional chemical extraction methods, the microbial extraction of chitosan is characterized by its low Mw, high solubility, and relatively high DD. This approach not only significantly reduces dependence on raw materials but also minimizes environmental pollution and production costs to the greatest extent possible [142,143]. Moreover, although chitosan exhibits broad-spectrum antibacterial activity against various microorganisms, its actual efficacy may vary significantly depending on specific microbial types, processing conditions, the DD of chitosan, and its solubility under different environmental conditions [144,145]. For instance, the primary antimicrobial mechanism of chitosan is the disruption of microbial cell membranes. Gram-positive bacteria have relatively simple cell membranes, while Gram-negative bacteria possess an additional protective layer outside their cell walls, making them more resistant to penetration and disruption by chitosan. This indicates that the antimicrobial activity of chitosan may vary depending on the type of microorganism [55]. Therefore, combining chitosan with other active substances such as drugs, metals, and natural compounds in nanosystems is a potential strategy for enhancing its antimicrobial activity [146]. However, when introducing multiple antimicrobial mechanisms, it is important to consider that chitosan may be incompatible with certain antimicrobial agents in different environments. It may also be affected by organic and inorganic compounds, which could alter its biocompatibility, low toxicity, and other biological activities, thereby limiting the potential for synergistic effects between chitosan and other antimicrobial agents [49,147]. Moreover, the optimization of DD is essential for the utilization of chitosan in various fields. Chitosan must have a DD of over 60% to be classified as commercial chitosan. High-purity chitosan (≥85%) has demonstrated significant effects in antibacterial and antioxidant applications, such as fruit and vegetable preservation, where it effectively inhibits bacterial growth and extends product shelf life [148,149]. Conversely, chitosan with a lower DD (60–85%) possesses favorable properties for medical applications, promoting the growth and differentiation of osteoblasts [150]. Therefore, the preparation of chitosan with varying degrees of acetylation is also a pressing issue that needs to be addressed. Moreover, chitosan is associated with several drawbacks, including limited solubility in non-acidic solutions, and susceptibility to changes in sensory characteristics, all of which restrict its further application. Therefore, future research on chitosan may focus on developing standardized production processes or exploring chitosan derivatives through chemical modification. For instance, carboxymethylation and quaternization can enhance the water solubility of chitosan and expand its antibacterial spectrum, improving its functionality across different pH environments [151,152]. Optimization of the Mw and DD of chitosan can ensure food safety while extending shelf life [153,154,155]. These modifications not only broaden the range of applications for chitosan but also address its limitations. However, it is important to note that with the widespread use of chitosan as an antimicrobial agent, bacteria may gradually adapt to its antimicrobial mechanism, potentially leading to the development of resistance [49]. Therefore, future research should focus on exploring the synergistic effects of chitosan with other antimicrobial mechanisms and its long-term efficacy in complex environments, which will be critical issues for the application of chitosan as an antimicrobial agent. Finally, it is crucial to note that although chitosan is widely used in clinical practice and is considered a safe biomaterial, there are a few reported cases of allergic reactions when using chitosan-based products for treatment. Therefore, before administering chitosan treatments, it is essential to thoroughly assess the patient’s allergy history, particularly regarding seafood allergies (e.g., shrimp and crab). This is due to the potential presence of trace protein residues during the processing of chitosan, which may trigger adverse reactions in individuals with seafood allergies. Additionally, appropriate measures, such as having epinephrine on hand, should be taken to manage potential allergic reactions during treatment [156].

Collectively, this article offers a comprehensive review of the structure of chitosan, preparation methods from different sources, antibacterial mechanisms, factors influencing its antimicrobial efficacy and latest applications across multiple fields, as well as an analysis of future developmental trajectories. Currently, the predominant method for sourcing chitosan remains chemical extraction; however, the utilization of microorganisms for chitosan extraction is expected to become the primary method for producing high-quality chitosan. Moreover, chitosan plays a crucial role in various industries such as biomedicine, food, pharmaceuticals, and cosmetics. However, its efficacy largely depends on key physicochemical properties including Mw and DD. Thus, it is imperative to extensively develop chitosan, a highly promising biomaterial, to make significant contributions to human health and well-being.

## Figures and Tables

**Table 1:** Chemical extraction method of chitosan source from marine crustaceans.

Source	Solvents and Conditions	Yield (%)	DD (%)	Ref.
Shrimp shells	Demineralization: ammonium-based ionic liquid; 110 °C; 24 h Deacetylation: 40% NaOH; 100 °C; 12 h	13.4	93	[17]
Crab shells	Demineralization: 2–4 M HCl; 28 ± 2 °C; 12–24 h Deproteinization: 1.5–3.5 M NaOH; 70 ± 0.5 °C; 1–3 h Deacetylation: 30–50% NaOH; (60–100) ± 0.5 °C; 1.5–4.5 h	13.29	84.2	[18]
Shrimp shells		16.93	89.73	

**Table 2:** Summary of in vitro antibacterial activity of chitosan composite/derivatives.

Chitosan Composites/Derivatives	Tested Microorganisms	Finding	Ref.
Chitosan nanoparticles with tripolyphosphate	*Pseudomonas* sp.	Exhibited antimicrobial effects against pathogens	[51]
Chitosan/phosphosilicate/Al₂O₃ nanosheets	*S. aureus*, *P. aeruginosa*, *C. albicans* and *A. niger*	Enhanced antibacterial activity against pathogens	[52]
Chitosan/CNPPV NCPs	*E. coli* and *S. aureus*	Demonstrated photo antimicrobial activity against bacterial resistance	[53]
Carboxymethyl chitosan doped with nickel and copper	*E. coli*, *Pseudomonas* *aeruginosa A*, *Pseudomonas aeruginosa B* and *Klebsiella* sp.	Enhanced antibacterial activity against pathogens and improved solubility.	[55]
N-alkylated chitosan	*E. coli*, *P. aeruginosa*, *S. aureus* and *Bacillus cereus*	Exhibited potent antimicrobial effects against pathogens	[56]
Quaternized chitosan nanofibers containing vanillin	*E. coli*, *S. aureus* and *C. albicans*	Collaborated with vanillin to extend the shelf life of fruits	[57]
Quaternized chitosan-dialdehyde cellulose composite sponges	*E. coli* and *S. aureus*	Possessed excellent hemostatic properties, used for wound care and emergency hemostasis	[58]
Quaternized chitosan	*C. albicans*, *Aspergillus flavus*, *E. coli* O157: H7 and *S. aureus*	Increased antimicrobial effects against pathogens	[59]

**Table 4:** Application of chitosan in water treatment.

Applications	Forms	Pollutants	Beneficial Effects	Ref.
Adsorption of heavy metal ions	Polyacrylic acid-grafted chitosan/biochar composites	Cu^2+^, Zn^2+^, Ni^2+^, Pb^2+^, Cd^2+^, Mn^2+^, Co^2+^, and Cr^3+^	Demonstrated the ability to adsorb heavy metal ions	[107]
	EDTA-carboxymethyl chitosan	Cu²⁺	Demonstrated the ability to adsorb Cu²⁺	[108]
	Phosphonium- crosslinked chitosan	Cr (VI)	Exhibited excellent adsorption capacity for wastewater treatment with a wider pH spectrum	[109]
Removal of microorganisms	Chitosan-modified clay	Prorocentrum	Treated harmful algal blooms in water bodies	[110]
	Chitosan lactate	Chlorella		[111]
	Inorganic salt chitosan-composite flocculant	Microalgae *Nannochloropsis* sp.		[112]

**Table 5:** Application of chitosan in agriculture (pest-resistant).

Applications	Forms	Antibacterial/ Insecticidal Substances	Tested Antibacterial Organisms/Pest	Beneficial Effects	Ref.
Pesticide carriers and pest resistance	Chitosan nanoparticles	Active nanosilver	*Colletotrichum coccodes*, *Aspergillus niger*, and *Pyricularia* sp.	Inhibited agricultural pathogens	[115]
		Spinosad and permethrin	*Drosophila melanogaster*	Improved insecticidal and bactericidal effects	[116]
		Carvacrol and linalool	*Tetranychus urticae*	Repelled and killed mites, and inhibited their spawning	[117]

**Table 7:** Applications of chitosan in food.

Application	Forms	Food	Beneficial Effects	Ref.
Food antibacterial matrix	Chitosan/nanocellulose composite film	Pomegranate seeds	Improved the freshness of food	[126]
	Chitosan-based membrane containing blueberry/blackberry extract	-	Maintained the freshness of vegetables and fruits	[127]
	Chitosan-based membrane containing mango leaf extract	Crushed nut	Reduced moisture content for vegetable and fruit preservation	[128]
Food additives	Chitosan nanoparticles loaded with Lactobacillus peptide	Orange juice	Reduced the number of bacteria in food	[130]
	Preservative paper containing 2-Hydroxyphenylacetic acid-g-chitosan	Honey peach	Increased the preservation effect of *Prunus persica*	[131]
	Synergistic composite of citric acid carboxymethyl chitosan and chlorine dioxide	Longan	Enhanced antiseptic of fruits and vegetables	[132]
	Quaternary dimethyl-(alkyl)-ammonium chitosan	Spinach	Reduced pathogens on vegetables	[133]

**Table 6:** Applications of chitosan in agriculture (plant growth and soil regulation).

Applications	Forms	Plant	Beneficial Effects	Ref.
Growth regulation in crops	Chitosan nanocomposites	Paddy	Promoted the growth and development of rice	[118]
	Copper (II) complex in chitosan/alginate microcapsules	Corn, barley and wheat	Promoted plant growth seed germination	[119]
	Modified oxidized chitosan	Wheat		[120]
Soil improvement	Chitosan-modified scrap iron-filing composite	-	Repaired the toxicity in contaminated soil	[121]
	Chitosan soil amendment	Lettuce	Increased crop yield	[122]
